# Dengue Reporter Virus Particles for Measuring Neutralizing Antibodies against Each of the Four Dengue Serotypes

**DOI:** 10.1371/journal.pone.0027252

**Published:** 2011-11-09

**Authors:** Kimberly Mattia, Bridget A. Puffer, Katherine L. Williams, Ritela Gonzalez, Meredith Murray, Emily Sluzas, Dan Pagano, Sandya Ajith, Megan Bower, Eli Berdougo, Eva Harris, Benjamin J. Doranz

**Affiliations:** 1 Integral Molecular, Inc., Philadelphia, Pennsylvania, United States of America; 2 Division of Infectious Diseases and Vaccinology, School of Public Health, University of California, Berkeley, California, United States of America; Institut Universitaire d'Hématologie, France

## Abstract

The lack of reliable, high-throughput tools for characterizing anti-dengue virus (DENV) antibodies in large numbers of serum samples has been an obstacle in understanding the impact of neutralizing antibodies on disease progression and vaccine efficacy. A reporter system using pseudoinfectious DENV reporter virus particles (RVPs) was previously developed by others to facilitate the genetic manipulation and biological characterization of DENV virions. In the current study, we demonstrate the diagnostic utility of DENV RVPs for measuring neutralizing antibodies in human serum samples against all four DENV serotypes, with attention to the suitability of DENV RVPs for large-scale, long-term studies. DENV RVPs used against human sera yielded serotype-specific responses and reproducible neutralization titers that were in statistical agreement with Plaque Reduction Neutralization Test (PRNT) results. DENV RVPs were also used to measure neutralization titers against the four DENV serotypes in a panel of human sera from a clinical study of dengue patients. The high-throughput capability, stability, rapidity, and reproducibility of assays using DENV RVPs offer advantages for detecting immune responses that can be applied to large-scale clinical studies of DENV infection and vaccination.

## Introduction

Dengue virus (DENV) is a member of the Flavivirus genus in the family *Flaviviridae* and consists of four distinct serotypes (DENV-1, DENV-2, DENV-3, and DENV-4), which are transmitted by *Ae. aegypti* and *Ae. albopictus* mosquitoes. DENV contains a single-stranded RNA genome of ∼10.7 kb that is translated as a single polyprotein and then cleaved into three structural (C, prM, and E) and seven nonstructural (NS1, NS2A, NS2B, NS3, NS4A, NS4B, NS5) proteins [1]. DENV is the most significant cause of arthropod-borne viral disease in humans, resulting in an estimated 50 million cases of dengue fever and over 450,000 cases of life-threatening dengue hemorrhagic fever/dengue shock syndrome (DHF/DSS) each year [2]. DHF/DSS can be fatal in up to 15% of affected individuals and is most commonly associated with sequential infection by different serotypes of the virus [3]. Although primary DENV infection may confer lifelong protection from re-infection with the same serotype, it only provides short-term protection from infection with additional serotypes [4], highlighting the need for a dengue vaccine that can confer persistent and simultaneous protection against all four serotypes of the virus. The development of such a vaccine continues to be a significant challenge, made more difficult by the lack of efficient and reliable screening methods for detecting and evaluating functional antibodies in human sera.

For over 40 years, the standard test for measuring DENV neutralization has been the Plaque Reduction Neutralization Test (PRNT), which measures the inhibition of viral infectivity (neutralization) as a reduction of plaque formation on a cell monolayer [5]. PRNT is not only labor intensive and technically complex, but requires the use of live virus, can vary based on the ability of a strain to form plaques, and is not readily adaptable to high-throughput analysis of large numbers of clinical or epidemiological samples. The limitations of PRNT have prompted the development of alternative means of detecting DENV neutralization using microplate ELISAs and flow cytometry-based assays [6], [7], [8]. These methods exceed the throughput of PRNT and demonstrate comparable sensitivity and specificity to PRNT when used to assay sera from patients after natural infection or vaccine administration. However, these detection methods also require the use of live DENV, do not facilitate genetic manipulation of infectious virions, and cannot always be readily adapted for large-scale measurements of neutralization [7].

Pseudo-infectious virions that express reporter genes have been used widely as tools to study several flaviviruses, including West Nile Virus (WNV) and tick-borne encephalitis virus [9], [10], [11], [12], [13]. Recently, a plasmid-based DENV RVP production system was developed as an easier and safer method for the genetic manipulation of infectious DENV virions and for measuring DENV infection and neutralization [14], [15]. Infection of permissive cells using DENV RVPs could be monitored directly by expression of a reporter gene that could be quantified using standard optical detection platforms. Initial studies primarily used the DENV RVP system to characterize temperature-dependent production of DENV-1 and DENV-2 RVPs using novel replicons, cell lines, and plasmid-based expression vectors. In the current study, we demonstrate the diagnostic utility of all four serotypes of DENV RVPs for measuring neutralizing antibodies, focusing on the suitability of DENV RVPs for large-scale, long-term clinical and epidemiological studies. DENV RVPs representing all four serotypes were tested in neutralization assays using both monoclonal antibodies and human sera, and data sets obtained using RVPs resulted in accurate neutralization titers comparable to PRNT. Neutralization titers using DENV RVPs were reproducible within experiments, across experimental days, between RVP production lots, and across two different laboratories. We also used DENV RVPs to accurately detect neutralizing antibodies in human serum samples from patients with primary or secondary DENV infections. Finally, we show evidence of the stability of DENV RVPs at 37°C and after long-term cryopreservation. Our results demonstrate that DENV RVPs provide a safe, rapid, and reproducible reagent for large-scale screening of sera for neutralizing antibodies, which can facilitate large-scale epidemiological studies and may help expedite dengue vaccine development.

## Results

### DENV RVPs are infectious and antigenically equivalent to live dengue virus

**Figure 1:**
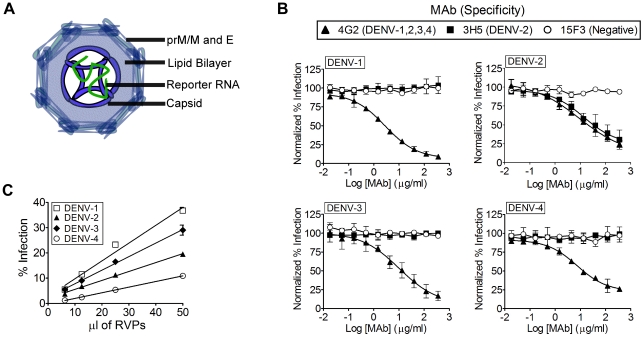
DENV RVPs are infectious and antigenically equivalent to live dengue virus. (**A**) Dengue Reporter Virus Particles (RVPs) are composed of the proteins capsid (C), premembrane/membrane (prM/M) and envelope (E) from defined DENV strains, and an RNA reporter genome made from a DENV genomic replicon. (**B**) Serial dilutions of DENV RVPs representing each serotype were used to infect Raji DC-SIGN-R cells. Forty-eight hours after infection, cells were analyzed for GFP expression by flow cytometry. Regression analysis shows a linear relationship (R^2^>0.98 for DENV-1; R^2^>0.99 for DENV-2; R^2^>0.99 for DENV-3; R^2^>0.99 for DENV-4) with the amount (µl) of RVPs used for infection (n = 4, error bars represent the standard deviation). (**C**) Neutralization assays using DENV RVPs were performed with serially diluted monoclonal antibodies 4G2, 3H5, or the control monoclonal antibody 15F3 for 1 hour prior to infection of Raji DC-SIGN-R cells. Forty-eight hours after infection, cells were quantified for GFP expression by flow cytometry. All neutralization results are shown normalized to the maximum (uninhibited) infection achieved, defined as 100% (n = 3, error bars represent the standard deviation). The infectious titers of the lots of RVPs used here, calculated based on the number of infected cells using an RVP input of 50 µl, are as follows: DENV-1: 773,325 infectious units/ml, DENV-2: 422,970 infectious units/ml, DENV-3: 347,292 infectious units/ml, and DENV-4: 353,995 infectious units/ml.

DENV RVPs are replication-incompetent virus particles that contain a sub-genomic DENV replicon encoding GFP, packaged by serotype-specific viral capsid (C), premembrane/membrane (prM/M), and envelope (E) proteins (**Figure 1A**). Upon entry into permissive cells, DENV RVPs express a GFP reporter gene for quantitative measurement of infection by flow cytometry (**Figure S1**). The production of DENV RVPs using a plasmid-based complementation approach has been described previously [14]. Using this approach, we developed DENV RVPs against all 4 dengue serotypes that are antigenically equivalent to commonly-used strains in DENV research and vaccine design: DENV-1 (WestPac), DENV-2 (S16803), DENV-3 (CH53489), and DENV-4 (TVP360). We used DENV RVPs to infect Raji DC-SIGN-R cells, a cell line commonly used for DENV studies that expresses DC-SIGN-R (CD209L), a C-type lectin adhesion cofactor shown to significantly enhance infection by enveloped viruses including DENV [16], [17], [18], [19]. Quantification of GFP signals were directly proportional to the amount of RVPs added to cells, demonstrating a linear correlation (DENV-1, R^2^>0.98; DENV-2, R^2^>0.99; DENV-3, R^2^>0.99; DENV-4, R^2^>0.99) between the extent of RVP infection and reporter gene expression (**Figure 1B**). We also tested a panel of cell lines commonly used in DENV studies, including BHK-21 and Vero cells, for DENV RVP infectivity. As expected, we observed varying degrees of infectivity, with the highest levels of infection occurring on cells expressing DC-SIGN and DC-SIGN-R (**Figure S2**). Because of their higher level of infection with DENV and their ease of use in flow cytometry assays, non-adherent Raji-DC-SIGN-R cells were used for subsequent experiments.

The primary humoral immune response generated against DENV is targeted to the prM/M and envelope proteins on the virion surface. In order to function as an alternative to live virus in neutralization assays, DENV RVPs must demonstrate antigenic equivalence to live DENV. To test the antigenicity of DENV RVPs, we assayed for antigen-specific neutralization of DENV-1, DENV-2, DENV-3 and DENV-4 RVPs using monoclonal antibodies (MAbs) that are cross-reactive against all four DENV serotypes (4G2), recognize only DENV-2 (3H5), or fail to neutralize DENV (15F3) [20], [21]. To obtain accurate neutralization titers, we used an RVP input of 25–50 µl since that gave both reproducible levels of infection of Raji DC-SIGN-R cells (10–20%) and resulted in signals within the linear range of detection. Cell infection rates outside the linear range (e.g. over 50% infection) could be achieved using optimized lots of RVPs (data not shown). Our results demonstrate that RVPs representing each of the four serotypes were neutralized using 4G2 and that only DENV-2 RVPs were neutralized by 3H5, as predicted (**Figure 1C**). The control DENV antibody (15F3) had no effect on infectivity for any serotype of DENV RVPs. These neutralization data suggest that, as designed, DENV RVPs appear antigenically authentic and can be used for the accurate detection of neutralizing antibodies against all four serotypes.

### DENV RVPs are stable and infectious following incubation at 37°C and prolonged cryopreservation

**Figure 2:**
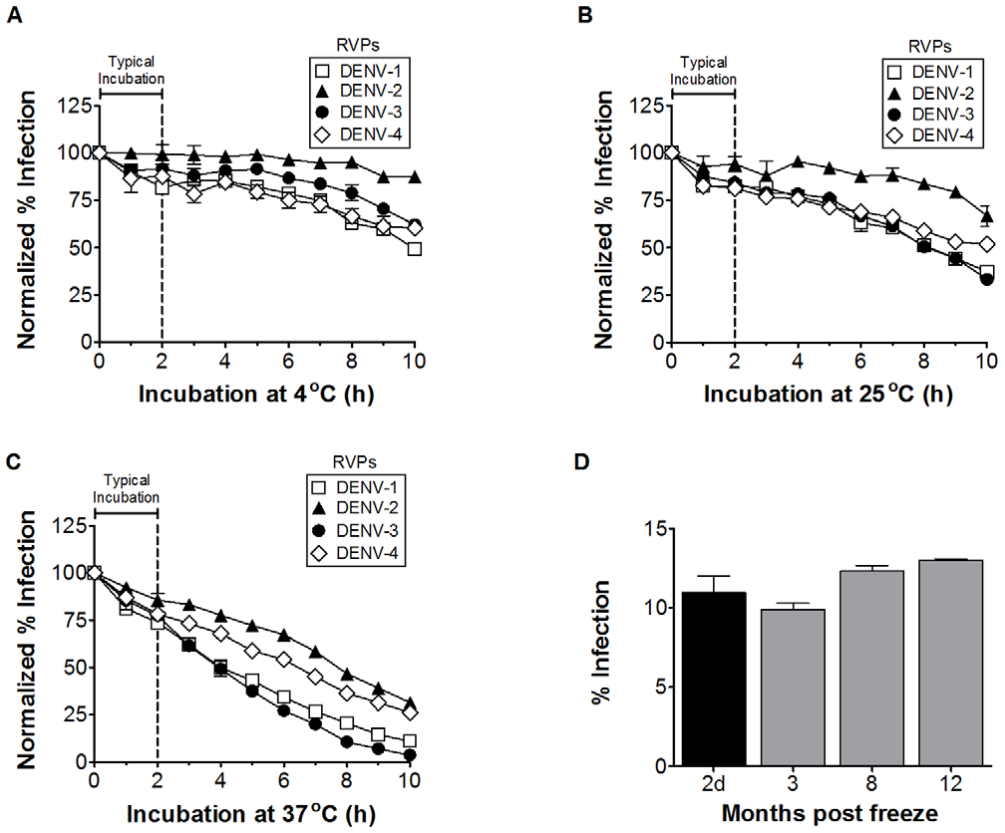
DENV RVPs are infectious under conditions commonly used in neutralization assays and following long-term cryopreservation. DENV RVPs representing all 4 serotypes were incubated at 4°C (**A**), 25°C (**B**), or at 37°C (**C**) for up to 10 hours, followed by infection of Raji DC-SIGN-R cells. Forty-eight hours post-infection, cells were analyzed for GFP expression by flow cytometry. At all temperatures, DENV RVPs remain highly infectious after a 2-hour incubation (a typical incubation period used in neutralization assays, vertical dashed line). (**D**) A single preparation of DENV-2 RVPs was aliquoted and frozen at −80°C from 2 days up to 1 year prior to infection of Raji DC-SIGN-R cells. Forty-eight hours after infection, cells were analyzed for GFP expression by flow cytometry. DENV RVPs showed little or no substantial loss of infectivity after long-term cryopreservation (n = 2, error bars represent the range).

The potential application of DENV RVPs to large-scale studies of human sera requires understanding the limitations of their use in practice. The ability to detect neutralization of DENV can be limited by the stability of the virus under commonly used experimental conditions, including incubation periods of up to several hours with neutralizing antibodies or serum and then with cells. To monitor the stability of DENV RVP preparations during typical infection assays, we incubated DENV RVPs of all four serotypes at 4°C, 25°C, or 37°C for up to 10 hours, followed by infection of Raji DC-SIGN-R cells (**Figure 2A–C**). DENV RVPs displayed high levels of infectivity after a 2 hour incubation at all temperatures and maintained significant infectivity even after extended incubation times at 4°C and 25°C. These results suggest that RVPs can withstand typical incubation temperatures (4°C to 37°C) and time periods (e.g. 1 to 2 hours) often used in neutralization assays.

The ability to prepare and store single lots of DENV RVPs for repeated experimentation can reduce variability associated with using different batches of reagents in large-scale studies where repeated measurements of infectivity over extended periods of time are required. To assess the stability of cryopreserved DENV RVPs, we assayed the infectivity of a single lot of DENV RVPs that was stored at −80°C from 2 days up to 12 months. DENV RVPs cryopreserved for up to 12 months demonstrated similar infectivity to DENV RVPs cryopreserved for 2 days (**Figure 2D**), suggesting that DENV RVPs can be a reliable reagent even for prolonged experiments. Taken together, our results demonstrate that DENV RVPs exhibit desirable stability characteristics for a reagent that could be used in long-term dengue studies.

### DENV RVPs can be used to derive reproducible antibody neutralization titers

**Figure 3:**
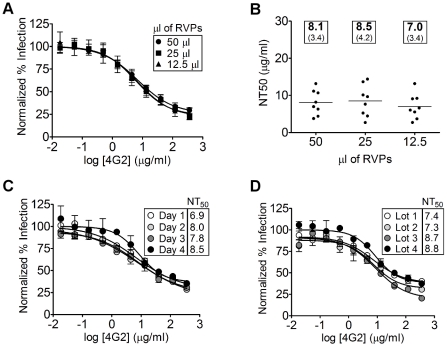
DENV RVPs can be used to derive reproducible antibody neutralization titers. (**A**) Serial dilutions of a single lot of DENV-2 RVPs were pre-incubated with the monoclonal antibody 4G2 at room temperature for 1 hour followed by infection of Raji DC-SIGN-R cells. Forty-eight hours after infection, cells were analyzed for GFP expression by flow cytometry (n = 8 for each dilution, error bars represent the standard deviation). (**B**) NT_50_ values for each replicate for the indicated RVP input were calculated and plotted (the bar represents the mean NT_50_, boxes show the mean and standard deviation for each RVP dilution tested). The mean NT_50_ values for each volume of RVPs tested for neutralization were not statistically different (ANOVA, p>0.05). (**C**) A single lot of DENV-2 RVPs was used for independent neutralization experiments performed on four different days in duplicate (mean and range shown). Mean NT_50_ values are not statistically different (ANOVA, p>0.05). (**D**) Independently derived lots of DENV-2 RVPs were used for neutralization experiments performed in duplicate (mean and range shown). Mean NT_50_ values are not statistically different across different lots (ANOVA, p>0.05).

Reproducible measurement of neutralization across RVP batches, test plates, and experimental days is critical for large-scale use and long-term studies. While reagent manipulations can be inherently variable from experiment to experiment, batch-specific factors should not affect neutralization titers. For example, excess antigen in the form of free E protein, subviral particles, or defective interfering virions could potentially bind to neutralizing antibodies to give an aberrant NT_50_ neutralization value (i.e., more antibody would be required to inhibit a given amount of infectious DENV RVPs) [13]. To test the reproducibility of neutralization measurements, we performed replicate neutralization assays using the monoclonal antibody 4G2 and three dilutions of a single lot of DENV-2 RVPs. RVPs were reproducibly neutralized by 4G2 (**Figure 3A**) and 50% neutralization titers were not statistically different, independent of RVP antigen input when in the linear range of detection (**Figure 3B**, ANOVA, p>0.05). Similar results were obtained with DENV1, DENV-3, and DENV-4 RVPs (**Figure S3**). These data suggest that DENV RVP preparations obey the laws of mass action [22], do not contain interfering excess antigen, and thus can be used to determine accurate neutralization titers. In addition, we observe similar NT_50_ values for independent neutralization experiments performed on four different days (**Figure 3C**) and across independently derived lots of all four serotypes of RVPs (**Figure 3D** **and Figure S3**), highlighting the reproducibility and reliability of RVPs for neutralization assays.

### Human sera neutralize DENV RVPs in a serotype-specific manner

**Figure 4:**
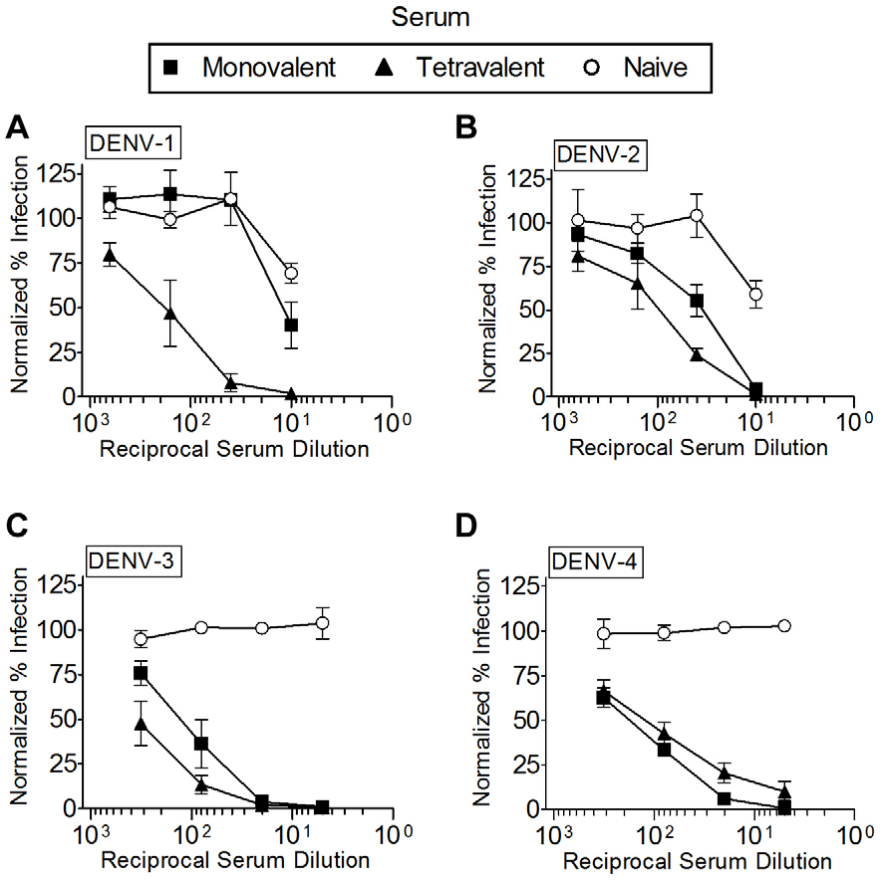
Neutralization of DENV RVPs using WHO human reference sera. DENV RVPs representing each of the four serotypes were pre-incubated for 1 hour with a WHO reference panel of human primary sera reactive to each RVP serotype (monovalent, squares: anti-DENV-1 serum in **A**; anti-DENV-2 in **B**; anti-DENV-3 in **C**; anti-DENV-4 in **D**) or reactive to all serotypes (tetravalent, triangles in all four panels). Naïve serum was used as a negative control. After incubation, DENV RVPs were used to infect Raji DC-SIGN-R cells. Forty-eight hours after infection, cells were quantified for GFP expression by flow cytometry (n = 3, error bars represent the standard deviation).

To be useful as a detection reagent for large-scale clinical dengue studies, DENV RVPs must be able to identify the presence of neutralizing antibodies in human serum. To test this, we used DENV RVPs in neutralization assays with a standardized WHO reference serum panel derived from naturally infected patients that has been used by laboratories throughout the world to validate PRNT [23]. This panel consists of sera reactive against each of the DENV serotypes, one tetravalent serum reactive against all the serotypes, and one non-reactive control serum. Similar to our MAb neutralization results, all four serotypes of DENV RVPs were completely neutralized by the tetravalent serum (**Figure 4**). Homotypic WHO serum against DENV-2, DENV-3 and DENV-4 demonstrated serotype specific neutralization. Monotypic serum used against each heterologous serotype did not display any significant neutralizing activity (**Table 1**, NT_50_ values), consistent with the observed antigenic specificity of RVPs. The WHO DENV-1 serum poorly neutralized DENV-1 RVPs (**Figure 4** **and** **Table 1**), but this result is consistent with previously reported PRNT values for this weakly neutralizing serum [23]. In contrast, human serum from a patient with a primary DENV-1 infection strongly and specifically neutralized DENV-1 RVPs (**Table 2**). As expected, control (naïve) serum failed to neutralize any DENV RVPs until non-specific inhibition was observed at the highest concentration tested. To further demonstrate the reproducibility of data derived using DENV RVPs, the same WHO serum-based neutralization assays were independently performed in two different laboratories (IM and UCB). NT_50_ values obtained in both laboratories were not statistically different (**Table 1** **, RVPa and RVPb**) (unpaired t-tests, p>0.05), suggesting that DENV RVPs can be used to derive serotype-specific and reproducible neutralization titers using human sera.

**Table 1:** Comparison of neutralization titers for human sera obtained by PRNT or RVPs.

		DENV-1 (WestPac)			DENV-2 (S16803)			DENV-3 (CH53489)			DENV-4 (TVP360)		
Serum		PRNT	RVPa	RVPb	PRNT	RVPa	RVPb	PRNT	RVPa	RVPb	PRNT	RVPa	RVPb
**DENV-1**	NT_50_	**24**	**<10**	**18**	18	12	10	14	<10	<10	11	<10	<10
	n, STDEV	32, 24	3, –	3, 15	32, 48	3, 6	3, 0.3	31, 18	3, –	3, –	31, 4	3, –	3, –
**DENV-2**	NT_50_	<10	<10	<10	**57**	**42**	**32**	13	<10	<10	11	<10	<10
	n, STDEV	31, –	3, –	3, –	32, 63	3, 8	3, 3	31, 19	3, –	3, –	31, 6	3, –	3, –
**DENV-3**	NT_50_	21	14	<10	<10	26	<10	**187**	**144**	**79**	11	<10	18
	n, STDEV	32, 44	3, 0.4	3, –	31, –	3, 3	3, –	32, 207	3, 54	3, 28	30, 8	3, –	3, 14
**DENV-4**	NT_50_	<10	<10	<10	<10	20	<10	10	<10	<10	**135**	**164**	**92**
	n, STDEV	32, –	3, –	3, –	32, –	3, 4	3, –	31, 5	3, –	3, –	32, 134	3, 44	3, 31
**DENV-1234**	NT_50_	**577**	**177**	**211**	**297**	**101**	**154**	**357**	**310**	**314**	**129**	**88**	**165**
	n, STDEV	32, 475	3, 44	3, 8	32, 313	3, 34	3, 55	32, 312	3, 24	3, 8	33, 146	3, 18	3, 63
**Control**	NT_50_	ND	<10	ND	ND	<10	ND	ND	<10	<10	ND	<10	<10
	n, STDEV	–	3, –	–	–	3, –	–	–	3, –	3, –	–	3, –	3, –

NT_50_ neutralization titers for sera were obtained using DENV RVPs or conventional PRNT with live virus and are expressed as reciprocal serum dilutions at which viral infection was inhibited by 50%. All reference sera were obtained from the National Institute of Biological Standards and Controls (NIBSC). RVPa and RVPb values were obtained from independent experiments performed in different laboratories (IM and UCB) and were compared to PRNT values obtained in an independent study conducted by the WHO for validation testing of PRNT for DENV clinical studies [23]. All sera were tested at least three times. Mean NT_50_ values using PRNT and RVPs were not statistically different (ANOVA, p>0.05). ‘<10’ (non-neutralizing) indicates a calculated NT_50_ value of <10 or the failure of the sera to neutralize by at least 50%. NT_50_ values <10 were calculated as 10.0 for statistical analysis. ND = not determined.

**Table 2:** Application of DENV RVPs to clinical serum samples.

	DENV-1 (WestPac)				DENV-2 (S16803)				DENV-3 (CH53489)				DENV-4 (TVP360)			
Serum	3 m	6 m	12 m	18 m	3 m	6 m	12 m	18 m	3 m	6 m	12 m	18 m	3 m	6 m	12 m	18 m
DENV-1 (DF)	**113**	**128**	**449**	**245**	<10	<10	<10	<10	47	48	44	39	42	45	62	133
DENV-1 (DHF)	**361**	**264**	**274**	**208**	11	<10	10	<10	52	66	61	42	73	79	29	<10
DENV-2 (DHF)	<10	<10	25	28	**104**	**152**	**658**	**429**	21	22	49	42	41	16	59	29
DENV-3 (DF)	15	<10	46	57	<10	25	30	26	**445**	**578**	**705**	**586**	132	54	52	80
DENV-3 (DHF)	28	<10	<10	15	39	<10	<10	<10	**1005**	**893**	**824**	**512**	<10	57	41	44
DENV-2 (secondary)	225	201	ND	342	179	130	ND	158	162	162	ND	152	<10	37	ND	<10

Longitudinal serum samples taken at 3, 6, 12 and 18 months post-symptom onset from two primary DENV-1, one primary DENV-2, two primary DENV-3, and one secondary DENV-2 infection were obtained from naturally infected patients in Nicaragua. The serum representing secondary infection was derived from a patient that was last infected by DENV-2, as determined by RT-PCR and virus isolation. Each serum was used in neutralization assays against each DENV serotype. ‘<10’ (non-neutralizing) indicates failure of the serum to neutralize by at least 50%. Each sample was run once, in duplicate. DF, Dengue Fever; DHF, Dengue Hemorrhagic Fever. ND = not determined.

To quantitatively compare our results to PRNT, mean NT_50_ values using RVPs with human WHO sera were compared to data derived using PRNT with live DENV. PRNT NT_50_ values were derived from a worldwide validation study of PRNT reproducibility (**Table 1** **, PRNT**) [23]. We found that DENV RVP NT_50_ values were not statistically different from PRNT NT_50_ values for each of the sera tested (**Table 1**, ANOVA, p>0.05). Thus, excluding the WHO DENV-1 serum because of its weak general reactivity, DENV RVP experiments were accurate in identifying serotype-specific neutralizing activity against homotypic or tetravalent sera with no false negative titers (i.e., 14 out of 14 assays that were expected to be neutralizing for a particular serotype were identified as neutralizing to that serotype, bolded results in **Table 1**) and no false positive titers (i.e., 24 of 24 assays using heterotypic or control sera showed little to no neutralizing activity, non-bolded results in **Table 1**). These results confirm the utility of DENV RVPs for accurately identifying and quantifying neutralizing and serotype-specific antibodies in human sera.

### Application of DENV RVPs to Human Clinical Samples

To demonstrate the utility of DENV RVPs for clinical serum samples, we tested the ability of RVPs to measure serotype- specific neutralizing antibodies in individuals naturally infected with DENV. We obtained a panel of 24 longitudinal serum samples from six patients (representing five primary infections and one secondary infection, collected at 3, 6, 12, and 18 months post-symptom onset) participating in a clinical study on dengue at the Hospital Infantil Manuel de Jesus Rivera (HIMJR) in Managua, Nicaragua. The infecting serotype for each patient had been previously determined by RT-PCR and/or virus isolation using acute-phase sera. For each serum sample, we performed six-point neutralization assays, in duplicate, using RVPs representing each of the four DENV serotypes. Neutralization titers (NT_50_ values) calculated for each sample tested demonstrate that RVPs correctly identified the infecting serotype for each of the 5 primary serum samples (**Table 2** **and Figure S5**). Comparison of NT_50_ values across serotypes show titers 4–10 fold higher against the infecting serotype than the other three serotypes (**Table 2** and **Figure S4**). As expected, sera from the secondary DENV infection demonstrated strong neutralization titers against multiple DENV serotypes, with the highest titer presumably corresponding to the initial serotype of infection (“original antigenic sin” [24]). Examination of the samples longitudinally over time suggests that both high levels of homotypic neutralizing antibodies and low levels of heterotypic neutralizing antibodies were maintained over an 18 month period and could be accurately measured using DENV RVPs (**Table 2**).

## Discussion

The development of a plasmid-based production system for DENV RVPs [14] enables DENV RVPs to be produced at large-scale and relatively high titer for widespread applications. Here, we demonstrate the production of DENV RVPs that are antigenically equivalent to each of the four DENV serotypes, facilitating the detection of neutralizing activity in human sera. DENV RVPs can be used for reliable and rapid quantification of DENV infection using stability and reproducibility metrics required for large-scale applications such as dengue epidemiological studies and vaccine trials. DENV RVPs are stable under typical conditions used in neutralization assays and can be utilized to define infecting serotypes and quantify neutralization titers present in human sera. We demonstrate the utility of DENV RVPs for neutralization assays using both MAbs and human serum and show that RVPs perform with a high degree of similarity to the traditional PRNT method of detection.

PRNT has been the conventional method for detecting DENV neutralizing antibodies for over 40 years [5]. However, data from a multi-center validation of PRNT within and between laboratories showed a high degree of variability in neutralizing NT_50_ values for the same serum samples [23], [25]. The large variation associated with PRNT is due in part to manual manipulations, subjective visual assessment of plaques, and variability in infectivity using live DENV preparations. PRNT also cannot be used with many vaccine strains or clinical isolates of DENV that plaque poorly or with cell types that do not support plaque formation [26], [27]. To address some of these limitations, alternative assays for detecting DENV infection and neutralization have been developed, including microneutralization and flow cytometry [6], [7], [8]. These formats have several advantages over PRNT, including single-cell detection, non-requirement for plaque formation, and relatively rapid detection (2–5 days post-infection). However, these assays still rely on live infectious DENV and cannot always be readily adapted to large-scale, high-throughput detection of neutralizing antibodies [7].

Protective immunity against all four DENV serotypes is a requirement for a safe and effective vaccine because, in a process known as Antibody Dependent Enhancement (ADE), low affinity or low concentration antibodies are believed to increase infection rates by binding virus particles and targeting them to cells expressing the Fcγ receptor (FcγR) [28], [29], [30]. We expect that DENV RVPs will also be useful for measuring ADE *in vitro*, and future studies will focus on establishing an infectivity assay using DENV RVPs in conjunction with cells that express FcγR to monitor ADE.

Collectively, our results demonstrate that DENV RVPs provide a safe, fast, reliable, and quantitative platform for measuring DENV infectivity and for identifying neutralizing antibodies against all four DENV serotypes. The ability of DENV RVPs to accurately detect infecting serotypes in serum samples from patients with primary DENV infections highlights their utility for clinical applications. DENV RVPs may thus serve as an attractive alternative to existing methodologies, with particular application to large-scale studies such as DENV vaccine trials, epidemiologic surveillance, and high-throughput drug screening.

## Materials and Methods

### Ethics Statement

This study was approved by the Institutional Review Boards of the Nicaraguan Ministry of Health and the University of California, Berkeley. Parents or legal guardians of all subjects provided written informed consent, and subjects over 5 years of age provided assent.

### Plasmids, Cells Lines, and Reporter Virus Particles

Plasmids encoding DENV structural genes (CprME) and a DENV GFP replicon are described elsewhere [14]. The following cell lines were used in this study: 293TREx (Invitrogen), BHK21 clone 15 (Center for Vector borne Diseases, UC Davis), Vero (ATCC), Raji-DC-SIGN and Raji-DC-SIGN-R (kindly provided by Robert Doms). All cell lines were grown at 37°C at 5% CO_2_ in DMEM complete medium (DMEM with 10% FCS and 1% penicillin-streptomycin and 2 mM L-glutamine) except for Raji-DC-SIGN cells, which were grown in RPMI with 10% FCS and 1% penicillin-streptomycin and Raji-DC-SIGN-R cells, which were grown in RPMI with 20% FCS and 1% penicillin-streptomycin and 2 mM L-glutamine. DENV RVP production (DENV-1, Western Pacific 74; DENV-2, S16803; DENV-3, CH53489; DENV-4, TVP360) was performed in 293TREx cell lines as described [14] [31]. Supernatants containing RVPs were harvested, passed through 0.45 µm filters, aliquoted, and stored at −80°C. For all experiments, DENV RVPs were rapidly thawed from cryopreservation in a 37°C water bath and placed on ice for use in neutralization assays.

### Monoclonal Antibodies and Human Sera

The following monoclonal antibodies (MAbs) were used: pan-DENV 4G2 (ATCC hybridoma [21]), anti-DENV-2 3H5 (ATCC hybridoma [21]), and anti-DENV-1 NS1 15F3 (ATCC hybridoma). The following convalescent sera from naturally-infected individuals were obtained from the UK National Institute for Biological Standards and Controls (NIBSC): monovalent DENV-1 (NIBSC Code 02/300), DENV-2 (NIBSC Code 02/296), DENV-3 (NIBSC Code 02/274), DENV-4 (NIBSC 02/298); negative control pre-bleed human sera (NIBSC Code 02/182), and tetravalent (DENV-1,2,3,4) human sera (NIBSC Code 02/186).

### DENV RVP Infection Assay

Raji-DC-SIGN and Raji-DC-SIGN-R cells were plated at a density of 40,000 cells per well in 96-well plates. DENV RVPs were diluted with RPMI complete medium and added to cells, followed by incubation at 37°C in 5% CO_2_. Forty-eight hours after infection, cells were fixed with 2% paraformaldehyde and analyzed by flow cytometry for GFP fluorescence. Typical infection assays used 25–50 µl RVPs and achieved infection rates of 10–20% (measuring the number of cells gated as GFP-positive divided by the total number of cells in the well). All neutralization results are shown normalized to the maximum (uninhibited) infection achieved. For BHK21 and Vero infection, cells were plated at a density of 30,000 cells per well in 96 well plates. DENV RVPs were diluted with DMEM complete medium and added to cells, followed by incubation at 37°C in 5% CO_2_. Forty-eight hours after infection, cells were trypsinized, fixed with 2% paraformaldehyde, and analyzed by flow cytometry for GFP fluorescence.

### DENV RVP Neutralization Assays

Neutralization assays were performed with MAbs or human serum samples. All serum samples were heat-inactivated at 56°C for 30 minutes, clarified by brief centrifugation, and filter sterilized (0.45 µm). DENV RVPs were pre-incubated in RPMI complete medium for 1 hour at room temperature with slow agitation with an equal volume of serially diluted antibodies (360 µg/ml to 0.0182 µg/ml pre-dilution, as measured based on the dilution of antibody prior to combining with RVPs) or 4-fold serially diluted sera (starting at 1∶5 or 1∶10 prior to combining with RVPs). Following incubation with either MAbs or serum, RVPs were transferred to a 96-well plate, and Raji DC-SIGN-R cells were added to each well at a density of 40,000 cells per well followed by incubation at 37°C in 5% CO_2_ for 48 hours. Cells were subsequently fixed in 2% paraformaldehyde and analyzed for the percentage of cells expressing GFP by flow cytometry (Guava Easycyte or Becton-Dickinson LSRII). The percent infection for each concentration of MAb or serum was calculated, normalized, and plotted as percent infection versus log_10_ of the MAb concentration or the reciprocal serum dilution. The data were fit to a sigmoidal dose-response curve using Prism (GraphPad Software, La Jolla, CA) to determine the titer of antibody that achieved a 50% reduction in infection (50% neutralization titer, NT_50_). Maximum infection was determined in the absence of antibodies.

### Clinical Serum Samples from Dengue Patients

Longitudinal serum samples were obtained from patients 6 months to 14 years of age who were admitted to the National Pediatric Reference Hospital, Hospital Infantil Manuel de Jesus Rivera (HIMJR, Managua, Nicaragua), and, after meeting the inclusion criteria, were enrolled in a dengue study [32], [33]. An individual was considered to be positive for DENV infection if any of the following four criteria were met: 1) DENV was isolated, 2) DENV RNA was demonstrated by RT-PCR, 3) an IgM capture ELISA was negative in the acute-phase serum sample and positive in the convalescent phase serum sample, or 4) antibody titers as measured by an inhibition ELISA demonstrated a ≥4-fold rise in paired acute and convalescent sera [34]. A primary DENV infection was defined by antibody titer of <1∶10 (acute) or antibody titer of <1∶2560 (convalescent), and a secondary DENV infection was defined by anti-DENV antibody titer of ≥1∶10 (acute) or ≥1∶2560 (convalescent), as determined by an inhibition ELISA. Daily serum samples were obtained during the acute phase, and additional samples were collected at 14 days, 3 months, 6 months, 12 months, and 18 months post-symptom onset.

## Supporting Information

Figure S1 **Quantification of DENV RVP infection by flow cytometry.** Representative flow cytometry plots from mock- and DENV RVP-infected Raji-DC-SIGNR cells. Percent RVP infectivity was calculated by measuring the fraction of GFP positive cells (upper right quadrant, pink) within the total number of live cells (blue+pink) gated from forward and side scatter plots. Infected cells above 50% can routinely be achieved, but infectivity <20% ensures linearity of infection and adherence to the law of mass action.
(TIF)

Figure S2 **DENV RVP infectivity of multiple cell lines.** DENV-2 RVPs were tested for infectivity with a panel of commonly used cell lines. Forty-eight hours after infection, cells were analyzed for GFP expression by flow cytometry (n = 2, error bars represent the range). BHK and Vero cells clearly demonstrated GFP-positive infected cells, but were less efficiently infected than cells containing the DC-SIGN or DC-SIGN-R cofactors.
(TIF)

Figure S3 **DENV RVPs can be used to derive reproducible antibody neutralization titers.** Three independent lots, each lot tested twice, of (**A**) DENV-1 RVPs (25 µl), (**B**) DENV-3 RVPs (3.1 µl), and (**C**) DENV-4 RVPs (25 µl) were pre-incubated with the monoclonal antibody 4G2 at room temperature for 1 hour followed by infection of Raji DC-SIGN-R cells. Forty-eight hours after infection, cells were analyzed for GFP expression by flow cytometry. Individual neutralization curves are shown for each replicate. Neutralization assays were performed using serial dilutions of three independent lots of (**D**) DENV-1 RVPs, (**E**) DENV-3 RVPs, and (**F**) DENV-4 RVPs, and mean neutralization curves are shown (n = 4–6 for each dilution, error bars represent the standard deviation). NT_50_ values for (**G**) DENV-1 RVPs (**H**) DENV-3 RVPs, and (**I**) DENV-4 RVPs for the indicated RVP input were calculated and plotted (bars represents the mean NT_50_, boxes show the mean and standard deviation for each RVP input tested).
(TIF)

Figure S4 **RVPs demonstrate serotype specificity using patient serum samples from primary and secondary DENV infections.** Six or twelve-month serum samples from naturally infected primary DENV-1 (**A**), primary DENV-2 (**B**), primary DENV-3 (**C**) or secondary DENV-2 (**D**) patients were serially diluted and incubated with RVPs from each of the four DENV serotypes for 1 hour at room temperature before infection of Raji DC-SIGN-R cells. Forty-eight hours post-infection, cells were quantified for GFP expression by flow cytometry. The dashed line depicts 50% neutralization (NT_50_) (n = 2, error bars represent the range).
(TIF)

Figure S5 **Reproducibility of RVP neutralization assays using human clinical serum.** NT_50_ neutralization titers for a human DENV-1 serum were obtained using DENV RVPs and areexpressed as mean reciprocal serum dilutions at which viral infection was inhibited by 50%. RVPa and RVPb values were obtained from independent experiments performed in different laboratories (IM and UCB), and mean NT50 values against DENV-1 RVPs or DENV-2 RVPs are not statistically different (unpaired t test, p>0.5), n = 3.
(TIF)
