# Supplementary figures and images for: Dengue Reporter Virus Particles for Measuring Neutralizing Antibodies against Each of the Four Dengue Serotypes
Source: PLoS One. 2011 Nov 9;6(11):e27252. doi: 10.1371/journal.pone.0027252 (PMC3212561; doi:10.1371/journal.pone.0027252)

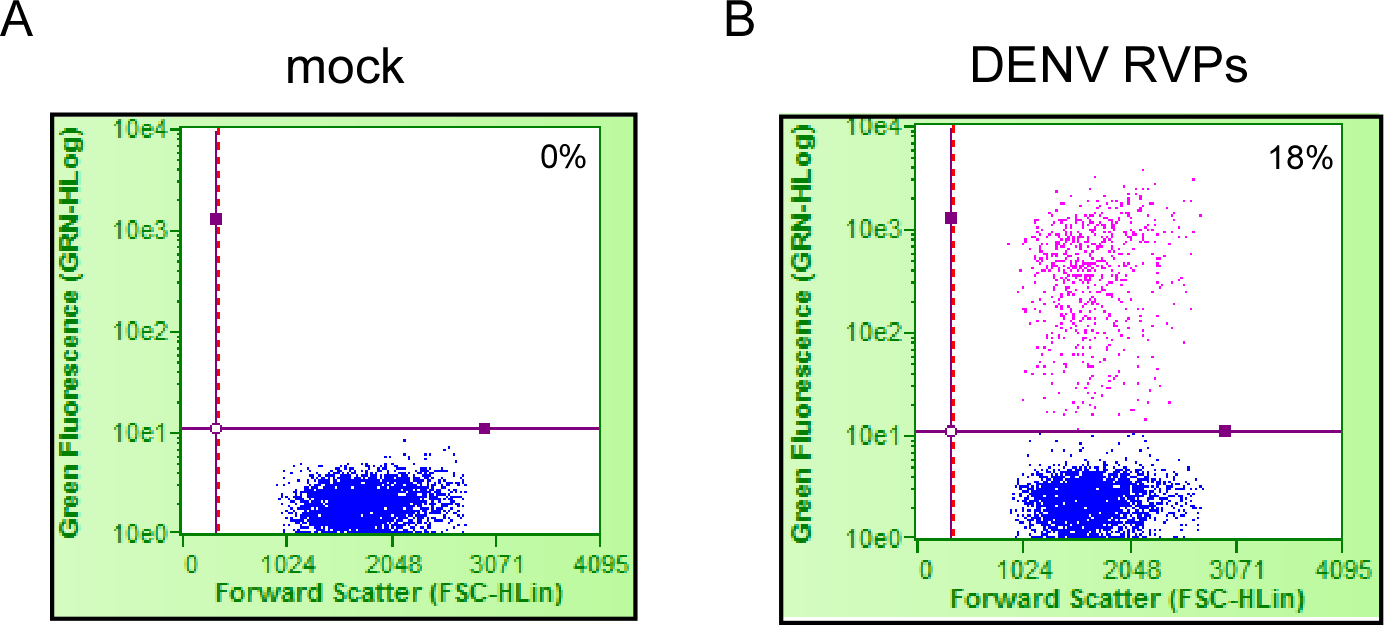

Supplement: Figure S1 — Quantification of DENV RVP infection by flow cytometry. Representative flow cytometry plots from mock- and DENV RVP-infected Raji-DC-SIGNR cells. Percent RVP infectivity was calculated by measuring the fraction of GFP positive cells (upper right quadrant, pink) within the total number of live cells (blue+pink) gated from forward and side scatter plots. Infected cells above 50% can routinely be achieved, but infectivity <20% ensures linearity of infection and adherence to the law of mass action. (TIF) [file pone.0027252.s001.tif]

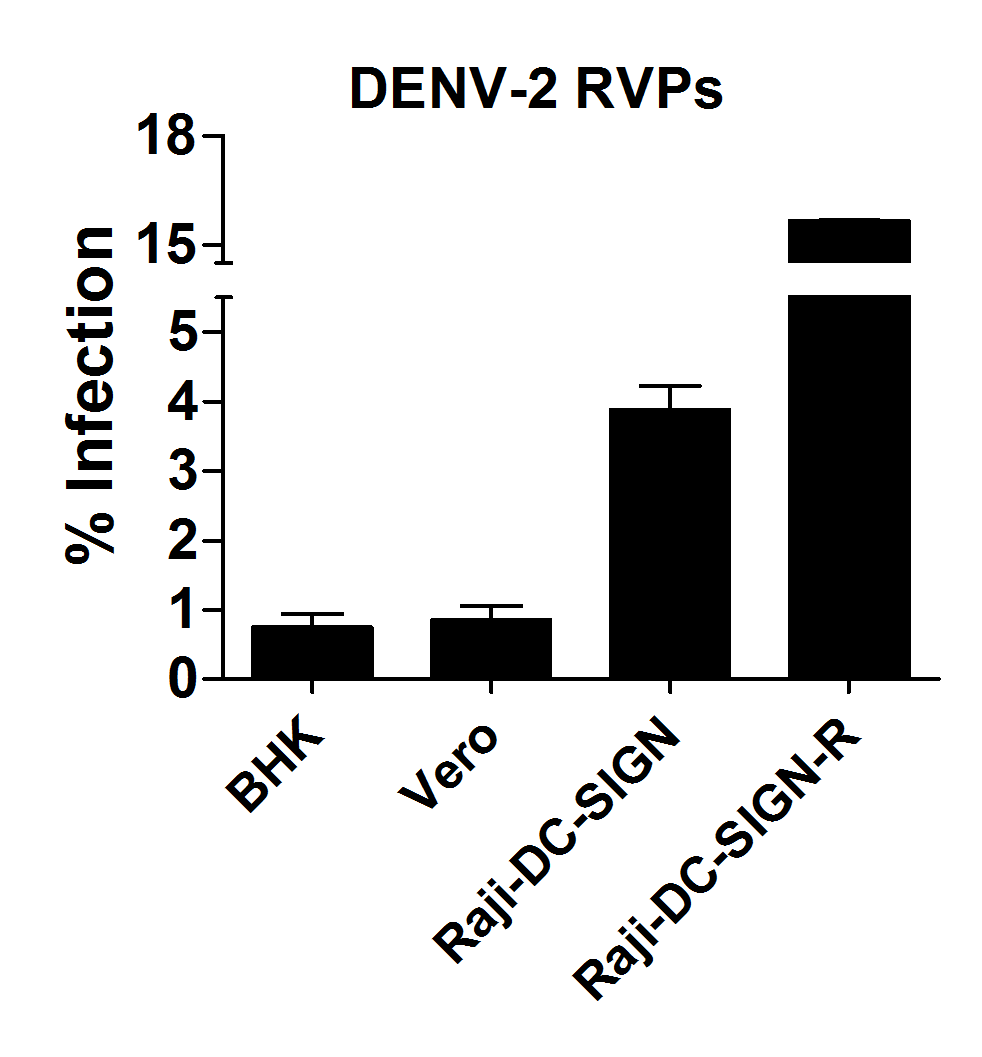

Supplement: Figure S2 — DENV RVP infectivity of multiple cell lines. DENV-2 RVPs were tested for infectivity with a panel of commonly used cell lines. Forty-eight hours after infection, cells were analyzed for GFP expression by flow cytometry (n = 2, error bars represent the range). BHK and Vero cells clearly demonstrated GFP-positive infected cells, but were less efficiently infected than cells containing the DC-SIGN or DC-SIGN-R cofactors. (TIF) [file pone.0027252.s002.tif]

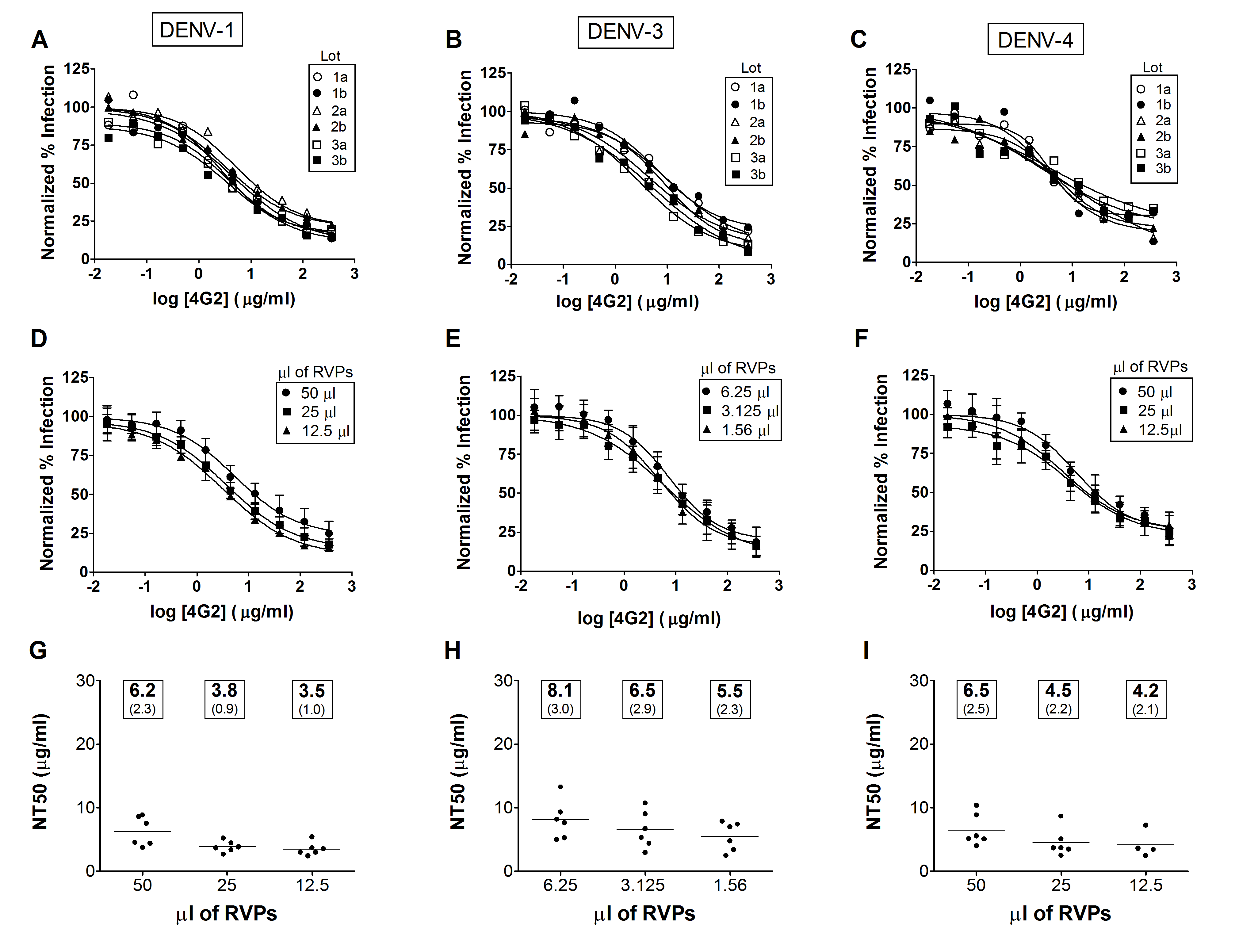

Supplement: Figure S3 — DENV RVPs can be used to derive reproducible antibody neutralization titers. Three independent lots, each lot tested twice, of (A) DENV-1 RVPs (25 µl), (B) DENV-3 RVPs (3.1 µl), and (C) DENV-4 RVPs (25 µl) were pre-incubated with the monoclonal antibody 4G2 at room temperature for 1 hour followed by infection of Raji DC-SIGN-R cells. Forty-eight hours after infection, cells were analyzed for GFP expression by flow cytometry. Individual neutralization curves are shown for each replicate. Neutralization assays were performed using serial dilutions of three independent lots of (D) DENV-1 RVPs, (E) DENV-3 RVPs, and (F) DENV-4 RVPs, and mean neutralization curves are shown (n = 4–6 for each dilution, error bars represent the standard deviation). NT50 values for (G) DENV-1 RVPs (H) DENV-3 RVPs, and (I) DENV-4 RVPs for the indicated RVP input were calculated and plotted (bars represents the mean NT50, boxes show the mean and standard deviation for each RVP input tested). (TIF) [file pone.0027252.s003.tif]

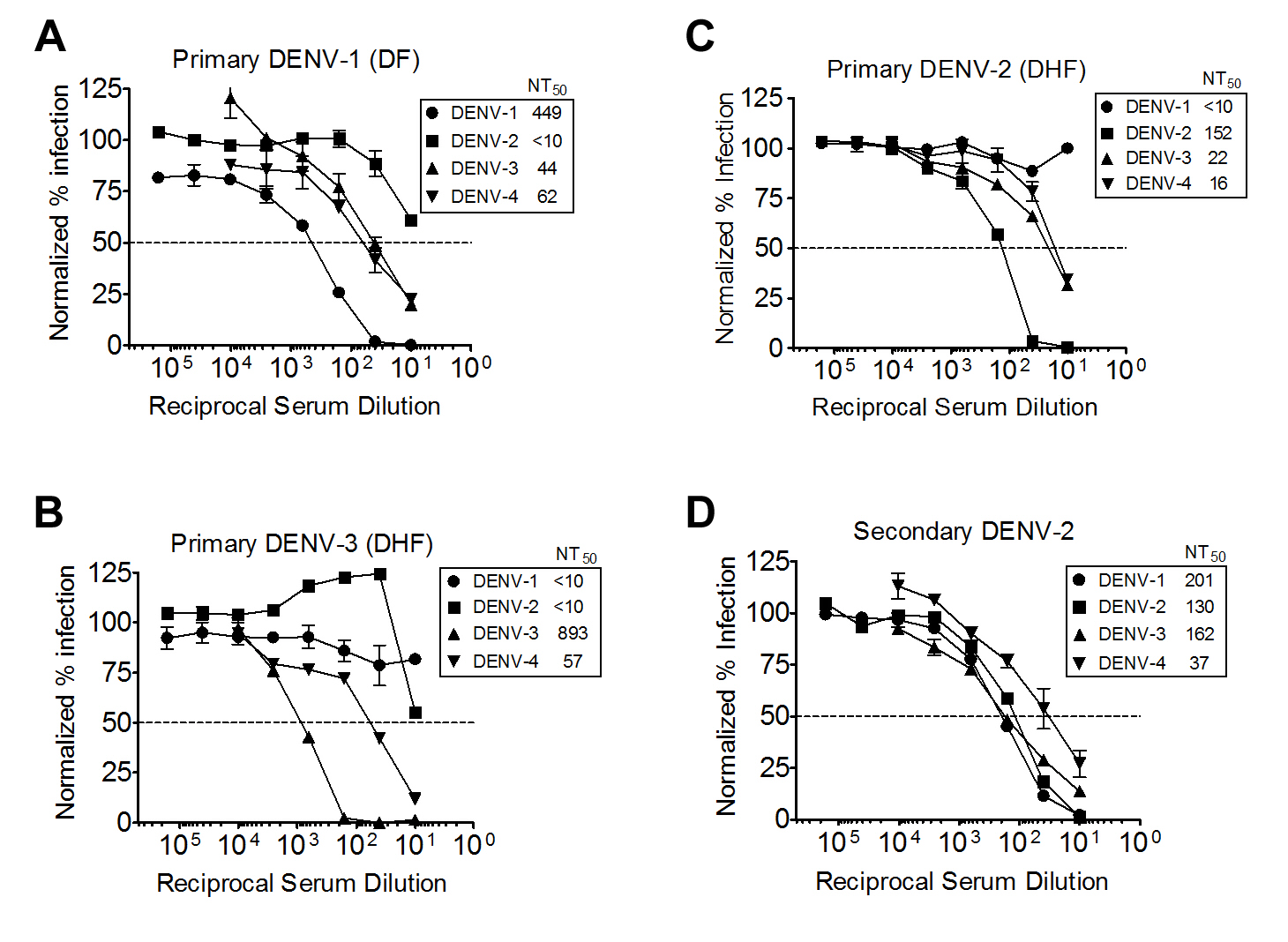

Supplement: Figure S4 — RVPs demonstrate serotype specificity using patient serum samples from primary and secondary DENV infections. Six or twelve-month serum samples from naturally infected primary DENV-1 (A), primary DENV-2 (B), primary DENV-3 (C) or secondary DENV-2 (D) patients were serially diluted and incubated with RVPs from each of the four DENV serotypes for 1 hour at room temperature before infection of Raji DC-SIGN-R cells. Forty-eight hours post-infection, cells were quantified for GFP expression by flow cytometry. The dashed line depicts 50% neutralization (NT50) (n = 2, error bars represent the range). (TIF) [file pone.0027252.s004.tif]

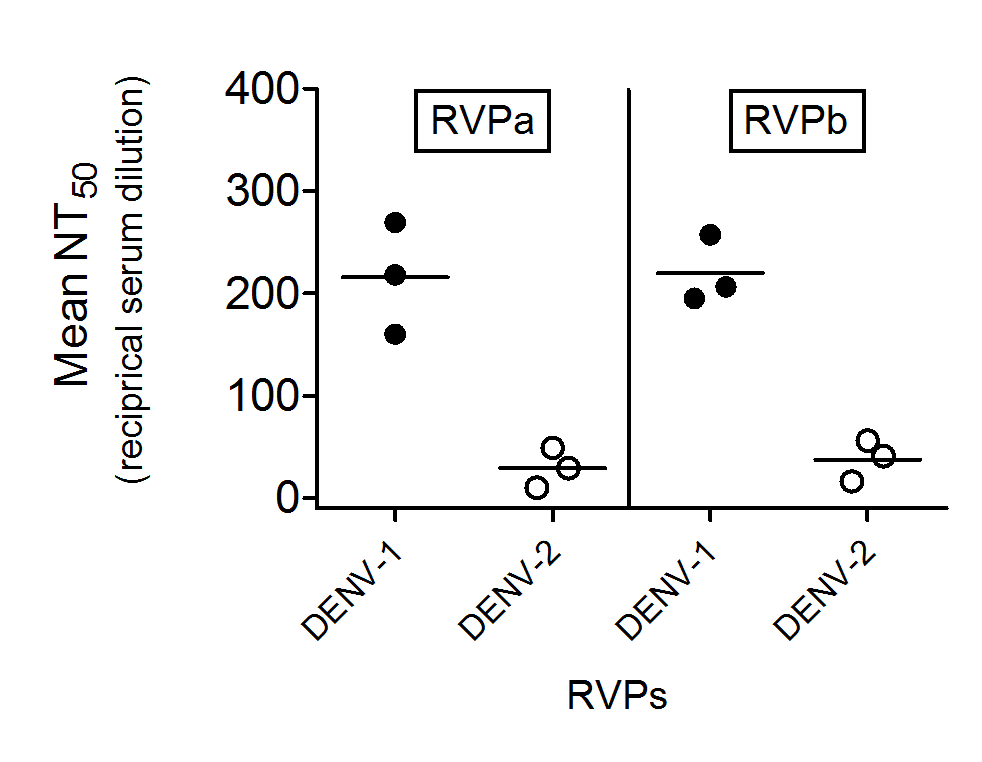

Supplement: Figure S5 — Reproducibility of RVP neutralization assays using human clinical serum. NT50 neutralization titers for a human DENV-1 serum were obtained using DENV RVPs and areexpressed as mean reciprocal serum dilutions at which viral infection was inhibited by 50%. RVPa and RVPb values were obtained from independent experiments performed in different laboratories (IM and UCB), and mean NT50 values against DENV-1 RVPs or DENV-2 RVPs are not statistically different (unpaired t test, p>0.5), n = 3. (TIF) [file pone.0027252.s005.tif]
